# Detection of *Echinococcus multilocularis* and other foodborne parasites in fox, cat and dog faeces collected in kitchen gardens in a highly endemic area for alveolar echinococcosis

**DOI:** 10.1051/parasite/2017031

**Published:** 2017-07-26

**Authors:** Marie-Lazarine Poulle, Matthieu Bastien, Yolan Richard, Émilie Josse-Dupuis, Dominique Aubert, Isabelle Villena, Jenny Knapp

**Affiliations:** 1 University of Reims Champagne-Ardenne, SFR Cap Santé, EA 3800 PROTAL 51092 Reims cedex France; 2 University of Reims Champagne-Ardenne, CERFE 08240 Boult-aux-Bois France; 3 French Institute for Fighting Zoonoses (ELIZ), Domaine de Pixéricourt 54220 Malzéville France; 4 University Hospital of Reims, Department of Parasitology-Mycology 51092 Reims cedex France; 5 University of Bourgogne Franche-Comté, Laboratory of Chrono-environment, UMR UFC/CNRS 6249 affi. INRA 25030 Besançon France; 6 University Hospital of Besançon, Department of Parasitology-Mycology 25030 Besançon France

**Keywords:** Carnivore faeces, Environmental contamination, *Vulpes vulpes*, *Felis catus*, *Toxocara* spp*.*, *Toxoplasma gondii*

## Abstract

*Echinococcus multilocularis, Toxoplasma gondii* and *Toxocara* spp. are foodborne parasites whose eggs or oocysts are spread in the environment via canid or felid faeces. They can cause infections in humans following the raw consumption of contaminated fruit or vegetables. In this study, their occurrence was investigated by quantitative polymerase chain reaction (qPCR) in 254 carnivore faeces deposited in 94 kitchen gardens of northeastern France that were sampled between two and six times from October 2011 to April 2013. Less than 25% of the sampled kitchen gardens contained more than 75% of the collected faeces. Of the 219 faeces that could be attributed to an emitter, cat accounted for 58%, fox for 32% and dog for 10%. *Echinococcus multilocularis* was detected in 35%, 11% and 7% of fox, dog and cat faeces, respectively, and *Toxocara* spp. in 33%, 12% and 5.5% of cat, fox and dog faeces, respectively. *Toxoplasma gondii* was detected in 2/125 cat faeces and 2/21 dog faeces. The 34 faeces that tested positive for *E. multilocularis* were found in only 19 out of the 94 sampled kitchen gardens, and the 40 faeces that tested positive for *Toxocara* spp. were found in 28 of them. Consequently, some kitchen gardens appeared particularly at risk of human exposure to foodborne parasites, including *E. multilocularis* responsible for alveolar echinococcosis (AE), which is a serious zoonosis. In endemic areas, kitchen garden owners should be informed about the zoonotic risk linked to carnivore faeces deposits and encouraged to set up preventive measures.

## Abstract (fr)

*Echinococcus multilocularis, Toxoplasma gondii* et *Toxocara* spp. sont des parasites transmissibles par l’alimentation dont les œufs ou oocystes sont répandus dans l’environnement avec les fèces de canidés ou félidés. Ils peuvent être responsables d’infections humaines suite à la consommation crue de fruits et légumes contaminés. Leur présence a été recherchée par qPCR dans 254 fèces de carnivores déposées dans 94 potagers du nord-est de la France, échantillonnés deux à six fois entre octobre 2011 et avril 2013. Moins de 25 % de ces potagers contenaient plus de 75 % des fèces collectées. Parmi les 219 fèces pour lesquelles l’émetteur a pu être identifié, 58 % étaient de chat, 32 % de renard et 10 % de chien. *Echinococcus multilocularis* a été détecté dans 35 %, 11 % et 7 % des fèces de renard, chien et chat respectivement et *Toxocara* spp. dans 33 %, 12 % et 5.5 % des fèces de chat, renard et chien, respectivement. *Toxoplasma gondii* a été détecté dans 2/125 fèces de chat et 2/21 fèces de chien. Les 34 fèces testées positives pour *E. multilocularis* ont été trouvées dans seulement 19 des 94 potagers échantillonnés et les 40 fèces testées positives pour *Toxocara* spp. dans 28 d’entre eux. En conséquence, certains potagers apparaissent particulièrement à risque d’exposition humaine aux parasites transmissibles par l’alimentation et, notamment, à *E. multilocularis* responsable de l’échinococcose alvéolaire qui est une grave zoonose. En zone d’endémie, les propriétaires de potagers devraient être informés du risque zoonotique lié au dépôt de fèces de carnivores et encouragés à prendre des mesures de prévention.

## Introduction

In recent years, the impact that foodborne parasites exert on food safety, food security, quality of life and livelihoods has begun to receive well-deserved global attention [46]. Humans can become infected through the ingestion of food, water or soil that has been contaminated with the infectious stage of a parasite, which is often released into the environment in animal faeces [9, 48]. Fresh fruit and vegetables have been identified as a vehicle of transmission for half of the 24 foodborne parasites that rank at the top of the multi-criteria ranking for risk management of foodborne parasites [46]. In this ranking, *Echinococcus multilocularis, Toxoplasma gondii* and *Toxocara* spp. rank third, fourth and twentieth, making them especially worthy of concern.

*Echinococcus multilocularis* is a helminth parasite that is responsible for human alveolar echinococcosis (AE), a rare but severe disease that is considered one of the most serious zoonoses in the Northern Hemisphere [51]. *Echinococcus multilocularis* eggs are excreted in the faeces of a definitive host, which in Europe is mainly the red fox (*Vulpes vulpes*) [13], although free-roaming domestic dogs (*Canis lupus familiaris*) and domestic cats (*Felis silvestris catus*) can also carry this parasite [7, 12, 52, 53]. *Toxoplasma gondii* is responsible for toxoplasmosis, a disease that is usually subclinical, but which can be fatal in immunosuppressed patients. Furthermore, transplacental transmission of *T. gondii* may lead to severe congenital infections [45]. Felids, and mostly the domestic cat, are the main definitive host of this parasite spreading its oocysts with their faeces [11]. Lastly, *Toxocara* spp. are responsible for toxocariasis, a zoonosis that is rarely diagnosed because it is generally asymptomatic, but that can occasionally lead to two main clinical syndromes in humans: ocular larva migrans and visceral larva migrans [39]. The species contributing most to environmental contamination by *Toxocara* spp. eggs in urban, suburban and rural areas are stray cat, dog and fox, respectively [36].

*Echinococcus multilocularis* eggs*,* embryonated *Toxocara* spp. eggs or sporulated *T. gondii* oocysts can be found in fruit and vegetables intended for human consumption; *Toxocara* spp. eggs have been detected in produce harvested from the soil in organic farms [24], and *T. gondii* and *E. multilocularis* DNA have been detected in fruit and vegetable samples taken from the environment [29, 30]. *Echinococcus multilocularis* and *Toxocara* spp. eggs, as well as *T. gondii* oocysts, are very resistant to adverse environmental conditions and can remain viable in the environment for years under optimal conditions of low temperatures and high humidity [2, 33, 54]. The inadvertent ingestion of eggs or oocysts from unwashed raw fruit and vegetables has been identified as a transmission risk for AE [23, 40], toxoplasmosis [1, 34] and toxocariasis [15, 20] in humans. Faecal deposition by infected carnivore hosts in cultivated plots devoted to growing produce could thus be a crucial amplifier of the risk of zoonotic diseases. To prevent pre-harvest contamination of raw fruit and vegetables, data about such deposits are needed.

The aim of this study was to provide information on the deposit of fox, dog and cat faeces, potentially contaminated with *E. multilocularis*, *T. gondii* or *Toxocara* spp., in privately owned kitchen gardens used to grow food for household consumption. The occurrence of foodborne parasites in these faeces was investigated by using molecular analysis to screen for parasitic DNA. The study was conducted in northeastern France, which is a high-risk area for human AE [41].

## Materials and methods

### Study area

This study was carried out in a rural area of the Ardennes department (49°25′ N, 4°50′ E) in northeastern France. This area was chosen because its cumulative incidence rate for human infection with AE during the 1982–2007 period was between 2.74 and 6.10, one of the highest reported in France [41]. The 1200 km^2^ study area was located in the southern part of the Ardennes, where data about the population dynamics of foxes and the transmission dynamics of *E. multilocularis* in this population were available [18, 19]. The study area was defined to encompass 19 villages in which a contact on site (mayors and/or other contacts) was in a position to introduce us to local gardeners, given that “having confidence in the field researcher” was a prerequisite in obtaining the authorisation to sample privately owned kitchen gardens. Depending on the size of the “trust network” of each contact, between 1 and 16 kitchen gardens were sampled per village.

The study area was characterised by small villages (most with fewer than 200 inhabitants) scattered in a landscape of cultivated fields, pastureland and woodland (consisting of oak *Quercus* spp., beech *Fagus sylvatica*, hornbeam *Carpinus betulus* and spruce *Picea abies*). Red fox density was about 3–4 foxes/km^2^ during the 2003–2006 period [19] and did not significantly vary from 2004 to 2015 [3]. The cat population was censused in a 460-ha area that encompassed two villages of the study area (Boult-aux-Bois and Briquenay); it reached 142 individuals (~30 cats/km^2^) during the 2008–2010 period [16], around ten times the abundance of the red fox population. The dog population did not exceed 30 individuals in these two villages (Poulle, pers. obs), and was therefore at least four times smaller than the cat population. The prevalence of *E. multilocularis* in the vulpine population in the study area was 53% during the 2001–2005 period [19], and 36% between 2005 and 2010 in the whole Ardennes department [5].

### Sampling

**Figure 1.**
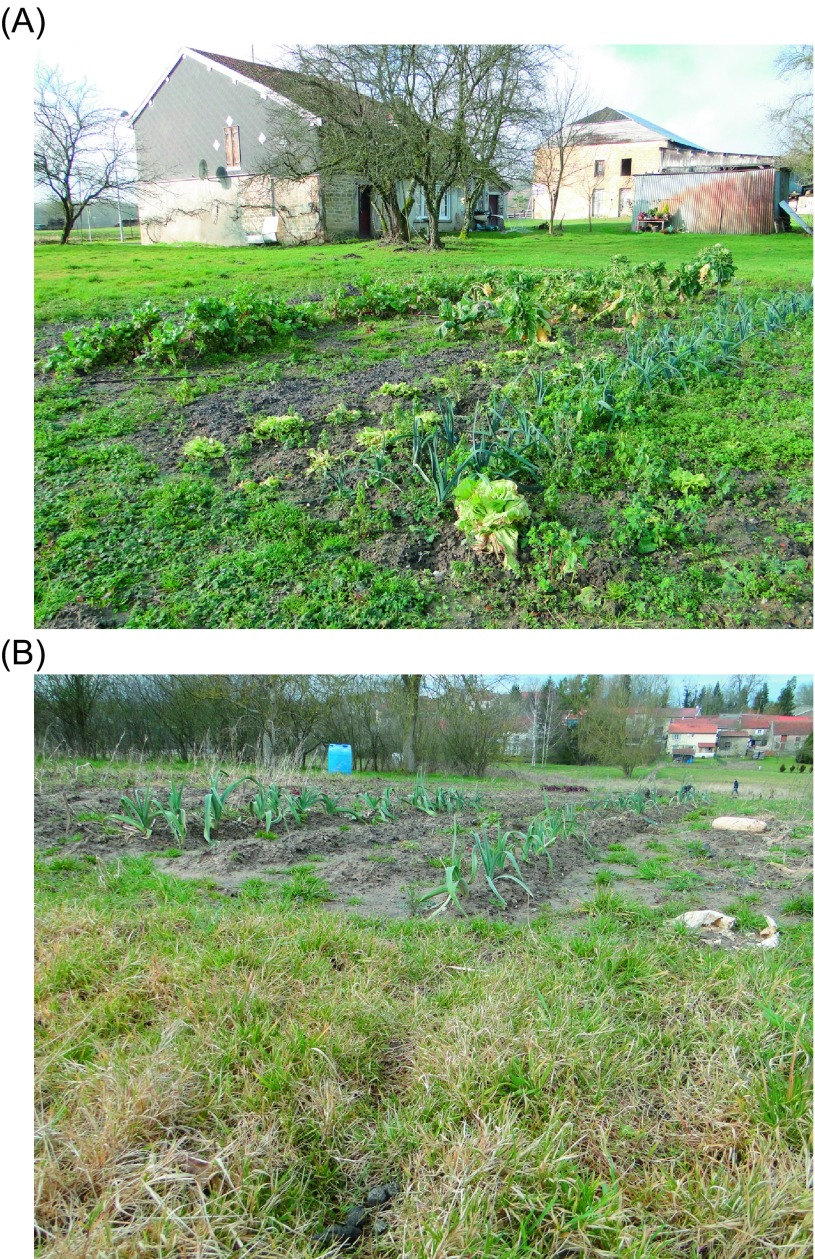
Two kitchen gardens in open access to carnivores and located inside (A) and outside (B) the village (at the forefront: a cat stool dropped close to the garden border).

From October 2011 to January 2012, 34 privately owned kitchen gardens in six villages in the study area were surveyed once a month to search for fox, dog and cat faeces. From February to April 2013, the sampling area was enlarged, including 16 out of the previously sampled 34 kitchen gardens (from 4 out of the 6 previous villages), plus 60 others from 13 different villages, making a total of 76 kitchen gardens for this period, and 94 privately owned kitchen gardens from 19 villages over the whole study period. These 94 kitchen gardens were only used for growing food (not for ornamental plants), providing vegetables and fruit such as lettuce, potatoes, carrots, leeks, cabbages, aromatic herbs, strawberries, etc. for one household. The size of the kitchen gardens – calculated by walking their perimeter with a Global Positioning System (GPS) device – was highly variable, ranging from 7 m^2^ to 2862 m^2^ for a mean of 360.5 ± 424.3 m^2^. The distance from a sampled kitchen garden to the closest dwelling or barn – also estimated by walking with a GPS device – ranged from 3 m to 202 m and averaged 24.7 ± 2.7 m (Fig. 1). Of the 94 sampled kitchen gardens, 32 (34%) were enclosed with a fence and were thus in principle not accessible to foxes, while the 62 others (66%) had open access to foxes, dogs and cats due to the absence of continuous fencing.

Sampling was conducted from October to April, i.e. outside the gardening period to avoid damaging seedlings. In that period, kitchen gardens are deserted, which increases the probability of carnivores visiting them, and plant cover is sparse, which makes faeces detection easier. Furthermore, *E. multilocularis* infection pressure in foxes is probably highest during the cold months of autumn and winter in northern France, as recently shown in Zurich (Switzerland) [38], thus increasing the likelihood of contamination at this time of year. It should be noted that parasite eggs or oocysts shed in kitchen gardens between October and April stand a good chance of still being present and viable during the following gardening period (May–September) due to the optimal conditions (low temperatures and high humidity) for these parasite stages during winter in the Ardennes.

We sampled 16 kitchen gardens six times (autumn–winter 2011–2012 and winter 2013), 75 three times (17 during autumn–winter 2011–2012, 58 during winter 2013), one five times, and two just twice. Each survey consisted of a walked transect that allowed for a visual scan of the whole kitchen garden surface area. The size of the sampled area was calculated per kitchen garden by multiplying its surface area with the number of times it was sampled. It ranged from 147 m^2^ to 17,172 m^2^, averaging 1411.9 ± 2136.8 m^2^ per kitchen garden, for a total sampled area size of 13.27 ha.

Fox, dog, cat or unidentified faeces were first discriminated visually in the field on the basis of shape and size, and then more accurately categorised by identifying the host species through faecal real-time PCR [26] (described below). The field researcher’s accuracy in discriminating cat, dog and fox faeces was verified by comparing the identification from morphological criteria to the identification of host species from molecular analysis. Faeces were collected with disposable gloves and put into separate labelled plastic bags. All collected faeces were decontaminated for 5 days at −80 °C and then stored at −20 °C before analysis.

### Molecular analyses

#### Copro-DNA extraction

DNA from copro-samples was purified using the QIAamp Fast DNA Stool Mini kit (Qiagen, Hilden, Germany). We used 0.5 g of each copro-sample and DNA purification was carried out as described in the manufacturer’s protocol.

#### PCR inhibitor control

The presence of PCR inhibitors was checked using a solution of 10^5^ spores/mL of *Geotrichum candidum* (strain no. 6560, from the Belgian Coordinated Collections of Micro-organisms (BCCM, http://bccm.belspo.be/about/ihE.multilocularis.php) as an internal control added to the copro-sample in the first step of the extraction protocol. Then, a qPCR was performed with an expected cycle threshold (Cq) value under 34 cycles, as previously described by Knapp et al. [25]. When inhibitors were detected, qPCR was performed a second time on copro-DNA diluted 1:10 and 1:100, respectively.

#### *E. multilocularis* and *Toxocara* spp. qPCR diagnosis

In order to detect the presence of *E. multilocularis* and *Toxocara* spp. parasites in the copro-samples, quantitative polymerase chain reactions (qPCRs) were performed on copro-DNA extracts, as previously described by Knapp et al. [25] for *E. multilocularis*, and Knapp et al. [26] for *Toxocara* spp. Briefly, a duplex qPCR was performed using TaqMan technology, with hydrolysis probes and TaqMan Gene Expression Master Mix 2X (Life Technologies, Foster City, CA, USA) to simultaneously detect the parasite and the PCR inhibitors (rrn-*E. multilocularis* primers and probe were associated with Geo primers and probe, and Tox primers and probe were associated with Geo primers and probe, Table 1). The qPCRs were performed with 45 cycles for the two parasites as previously described, in a 7500 Fast Real-Time PCR System (Applied Biosystems, Foster City, CA, USA). Results were analysed using 7500 2.0.5 software. A qPCR with Cq ≤ 45 cycles allowed us to determine the presence of parasites but qPCR results did not enable estimation of the amount of eggs or oocysts per faecal sample, and it was therefore not possible to estimate the amount of absolute contribution of fox, dog and cat to the contamination of gardens. Molecular analyses were performed at the Chrono-environment Laboratory, UMR 6249, UBFC, Besançon, France.

**Table 1.** Primers and hydrolysis probes used to detect *Echinococcus multilocularis*, *Toxoplasma gondii* and *Toxocara* spp. parasites, and carnivore host identification of copro-samples.

Targeted species	Gene	Sequence name	Nucleotide sequence	Reference
*Echinococcus multilocularis*	*rrnL*	rrn-Fwd	5′-CTGTGATCTTGGTGTAGTAGTTGAGATTT3′	[25]
		rrn-Rev	5′-GGCTTACGCCGGTCTTAACTC-3′	
		rrn-probe	5′-TGGTCTGTTCGACCTTTTTAGCCTCCAT-3′	
				
*Toxocara* spp.	*Cox1*	Toxo-Fwd	5′-AAAATAGCCAAATCCACACTACTACCA-3′	[26]
		Toxo-Rev	5′-GGTGTGGGACTAGTTGAACTGTGTA-3′	
		Toxo-probe	5′-CCCCATAGTCCTCAAAG-3′	
				
*Toxoplasma gondii*	*529-rep*	Tg-Fwd	5′-AGAGACACCGGAATGCGATCT-3′	[32]
		Tg-Rev	5′-CCCTCTTCTCCACTCTTCAATTCT-3′	
		Tg-probe	5′-ACGCTTTCCTCGTGGTGATGGCG-3′	
				
*Vulpes vulpes*	*CytB*	Vv-Fwd	5′-ACCTTCCCGCACCATCAAA-3′	[26]
		Vv-Rev	5′-TGTTGCAATCTGTAGAATAAGGCATA-3′	
		Vv-probe	5′-CTGCCTGATGGAACTTCGGGTCCC-3′	
				
*Canis l. familiaris*	*CytB*	Cf-Fwd	5′-CCACCCACTAGCCAAAATTGTT-3′	[26]
		Cc-Rev	5′-AAGTTCCATCAAGCAGAGATGTTAGA-3′	
		Cf-probe	5′-ATAACTCATTCATTGACCTCCCAGCGCC	
				
*Felis s. catus*	*CytB*	Fc-Fwd	5′-CCCTTCTAGGAGTCTGCCTAATCTT-3′	[26]
		Fc-Rev	5′-CGGTTATTGTGTCTGATGTGTAGTGT-3′	
		Fc-probe	5′-AAATCCTCACCGGCCTCTTTTTGGC-3′	
				
*Geotrichum candidum*	*CytB*	Geo-Fwd	5′-CACCGCCCGTCGCTAC-3′	[25]
		Geo-Rev	5′-AGAAAAGTTGCCCTCTCCAGTT-3′	
		Geo-probe	5′-TCAATCCGGAAGCCTCACTAAGCCATT-3′	

#### *Toxoplasma gondii* diagnosis

DNA extracts were subjected to a PCR targeting a specific repeated element of 529 bp [44] and performed using an iQ5 instrument (Bio-Rad, Hercules, CA, USA) as follows. A *Toxoplasma gondii*-specific target region (GenBank Accession No. AF487550) was amplified with a labelled TaqMan probe (5′6FAM-ACGCTTTCCTCGTGGTGATGGCG-3′TAMRA) and DNA oligonucleotide primers (5′-AGAGACACCGGAATGCGATCT-3′ and 5′-CCCTCTTCTCCACTCTTCAATTCT-3′) [32]. The amplification mixture was composed of 12.5 μL of 2× reaction mixture (Platinum Quantitative PCR SuperMix-UDG, Invitrogen, Carlsbad, CA, USA), 3.5 mM MgCl_2_, 0.5 μM of each oligonucleotide primer, 0.2 μM TaqMan probe, 1 μL of 1% bovine serum albumin to increase the PCR performance, and 5 μL of template DNA, for a final volume of 25 μL. The reaction mixture was initially incubated for 3 min at 50 °C to allow for uracil-N-glycosylase (UNG) activity. This first incubation was followed by a second incubation of 3 min 30 s at 95 °C to denature the DNA template, inactivate the UNG enzyme and activate the Platinum Taq DNA Polymerase. Samples were amplified as follows: 45 cycles of denaturation at 95 °C for 15 s, and annealing/extension at 60 °C for 1 min. Each sample was tested in duplicate. Negative controls were included from DNA extraction to PCR amplification steps, and each PCR run contained both negative and positive controls. No amplification results were systematically obtained for the negative control. A PCR with Cq ≤ 45 cycles allowed us to determine the presence of parasites. Molecular analyses were performed at the Laboratory of Parasitology and Mycology, EA3800 PROTAL, Reims University, France.

#### Host faecal test

The field researchers’ morphological identification of carnivore faeces was confirmed by a molecular host faecal test developed by Knapp et al. [26], using TaqMan probes and based on qPCR technology. Briefly, duplex qPCRs were performed to identify a stool as that of a red fox, dog or cat (Table 1) in the expected ranges of Cq values, as previously described.

#### Statistical analysis

Correlations between the number of faeces found and the sample sizes, and between the number of faeces testing positive for parasites and the total number of faeces found, were tested by the Spearman correlation test. The frequencies of faeces with positive qPCR results were given with their 95% confidence intervals and were compared using chi-square tests. A Fisher’s exact test was carried out when sample size was not sufficient. All statistical analyses were performed with R version 3.1.3.

## Results

A total of 85 faeces were collected from October 2011 to January 2012, and 169 faeces from February to March 2013, resulting in 254 faeces collected over the 2011–2013 period. Of the 254 faeces analysed, 194 (76.4%) were visually identified in the field from morphological criteria (size and shape), 191 (75.2%) provided usable DNA and 143 (56.3%) allowed for identification of the emitter species using qPCR analysis. Of the 104 faeces with a specific identification based both on morphological criteria and qPCR analysis, 19 out of 23 faeces (82.6%) and 54 out of 62 faeces (87.1%), respectively, attributed to fox and cat in the field were confirmed by qPCR analysis (Table 2). Thus, morphological criteria alone were considered reliable for the identification of cat and fox faeces. Consequently, the 50 faeces attributed to cat in the field but that did not permit qPCR identification of the emitter were considered as cat faeces, and the 26 faeces attributed to fox based on their morphological identification without molecular confirmation were considered as fox faeces. In contrast, as only 12 out of 19 (63.2%) faeces attributed to dog in the field were confirmed as dog stools by qPCR analysis (Table 2), morphological identification alone was not considered reliable enough to attribute faeces to dog. For that reason, only the 22 faeces identified as dog faeces by molecular analysis (among them some that were unidentified or misidentified in the field) were considered for further analysis. Of the 60 unidentified faeces in the field, 39 were identified by qPCR. In the 62 stools in which the Geotricum qPCR was inhibited, 40 could be identified by morphological and/or molecular analysis. Of these, 37 were attributed to cat, one to dog and two to fox. Cat faeces therefore accounted for almost all the identified faeces that had inhibitors.

**Table 2.** Concordance matrix between faeces identification based on morphological assessment and faeces identification based on molecular analysis (by qPCR). In grey cells: occurrence of faeces per species for which the morphological identification was concordant with the molecular analysis (e.g. 19 out of 23 faeces identified as fox faeces from morphological examination were confirmed in molecular analysis). In other cells: the number and percentage of faeces for which the morphological identification did not correspond with the molecular analysis. Percentages are given with their 95% confidence intervals.

	Fox by qPCR		Dog by qPCR		Cat by qPCR	
Fox	19/23	82.6% (61.2–95.0)	0/23	0	4/23	17.4% (4.9–38.8)
Dog	3/19	15.8% (3.4–39.6)	12/19	63.2% (38.3–83.7)	4/19	21.1% (6.0–45.5)
Cat	6/62	9.7% (3.6–19.9)	2/62	3.2% (0.4–11.1)	54/62	87.1% (76.1–94.3)

To sum up, 219 out of 254 (86.2%) of the collected faeces could be attributed to a species based on qPCR analysis and/or visual identification. Of these, 127 (58%) were cat faeces, 70 (32%) were fox faeces and 22 (10%) were dog faeces.

**Figure 2.**
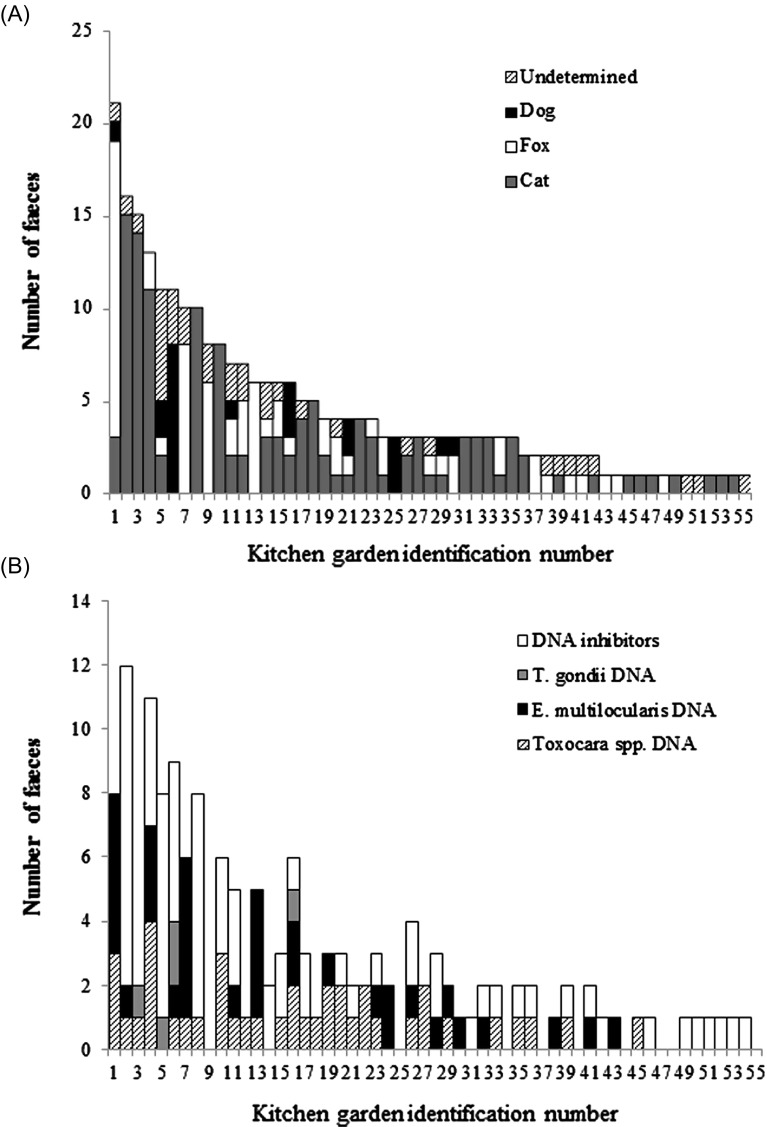
The number of carnivore faeces collected in the 55 (out of 94) sampled kitchen gardens in which at least one stool was collected; (A) the number of cat, fox, dog and unidentified faeces; (B) the number of faeces that yielded positive results for the detection of *Toxocara* spp., *Echinococcus multilocularis* or *Toxoplasma gondii*, as well as the number of faeces with DNA inhibitors.

No stools were found in 39 of the 94 sampled gardens (41.5%), whereas 1–21 stools were collected in the 55 others (Fig. 2A). The kitchen garden where the highest number of faeces were collected (21 faeces: 16 fox stools, 3 cat stools, 1 dog stool and 1 undetermined stool) was the farthest from buildings or barns (by a distance of 234 m). The 23 out of 94 kitchen gardens (24.5%) where at least four stools were found were the source of 191 out of 254 (75.2%) faeces. As might be expected, the number of faeces found per kitchen garden was significantly correlated with the surface sampled (*r* = 0.54, *p* < 0.0001). Of the 52 kitchen gardens where at least one identified carnivore stool was found, 35 (67%) had only cat, fox or dog faeces, 10 (19%) had both cat and fox faeces, 1 (2%) had both dog and fox faeces, and 6 (12%) had cat, dog and fox faeces (Fig. 2A). Of the 23 kitchen gardens that had only cat faeces, 8 (33%) were enclosed. There were four kitchen gardens with more than 10 cat faeces: these were characterised by the close proximity of a site where cats were fed (by the kitchen garden owners or their neighbours).

Of the 74 faeces that yielded positive qPCR or PCR results for the detection of parasite DNA, three tested positive for both *Toxocara* spp. and *E. multilocularis* (two fox stools and one cat stool) and two others tested positive for both *Toxocara* spp. and *T. gondii* (one cat stool and one dog stool), resulting in a total of 79 parasite DNA detections (Table 3). The occurrence of *Toxocara* spp. and *E. multilocularis* DNA did not significantly differ within the total sampling, with 20.7% and 18.3% of stools testing positive, respectively (Table 3, χ^2^ test = 0.22, *p* = 0.64). However, the occurrence of *Toxocara* spp. and *E. multilocularis* differed significantly (*p* < 0.001) between cat, fox and dog faeces that yielded qPCR results (Table 3). *Toxocara* spp. was the most frequently detected parasite in cat faeces (33.3%), while its occurrence was significantly lower in fox faeces (11.6%) and dog faeces (5.5%) (*p* < 0.001). In contrast, *E. multilocularis* was significantly more frequent in fox faeces (34.8%) than in cat faeces (7%) or dog faeces (11.1%) (*p* < 0.0001). *Toxoplasma gondii* was detected in 2 out of 125 cat faeces and 2 out of 21 dog faeces and was not detected in fox faeces (Table 3). The two dog faeces that tested positive for *T. gondii* PCR were collected within a one-month interval in the same enclosed kitchen garden. They originated from the same privately owned dog. Of the total eight faeces collected from this dog, one was contaminated with both *T. gondii* and *Toxocara* spp, one was contaminated with *T. gondii* and one was contaminated with *E. multilocularis.*

**Table 3.** Occurrence of cat, fox, dog and unidentified faeces collected in kitchen gardens that yielded positive PCR results for the detection of *Echinococcus multilocularis*, *Toxocara* spp. or *Toxoplasma gondii* DNA as compared to the total number of faeces that yielded PCR results for the given emitter and parasite (i.e. faeces without inhibitors). Percentages are given with their 95% confidence intervals.

	*E. multilocularis*		*Toxocara* spp.		*Toxoplasma gondii*	
Cat	6/86	7% (2.6–14.6)	31/93	33.3% (27.0–48.6)	2/125	1.6% (0.1–5.7)
Red fox	24/69	34.8% (23.5–47.6)	8/69	11.6% (5.1–21.6)	0/28	0%
Dog	2/18	11.1% (1.4–34.7)	1/18	5.5% (0.1–27.3)	2/21	9.5% (1.2–30.4)
Unidentified	2/13	15.4% (1.9–45.4)	0/13	0%	1/25	4.0% (0.1–20.3)
Total	34/186	18.3% (13.0–24.6)	40/193	20.7% (15.2–27.1)	5/199	2.5% (0.8–5.7)

In 7 of the 55 kitchen gardens where at least one stool was found, all the stools had inhibitors, resulting in an absence of qPCR results (Fig. 2B). The 79 detections of parasite DNA originated from 36 of the 94 sampled kitchen gardens (38.3%). The number of parasite detections was correlated to the number of faeces collected per kitchen garden (*r* = 0.55, *p* < 0.0001). The 34 faeces that tested positive for *E. multilocularis* were collected in 19 of the 94 sampled kitchen gardens (20.2%), while 28 of the 94 sampled kitchen gardens (29.8%) contained the 40 faeces that tested positive for *Toxocara* spp. (Fig. 2B).

## Discussion

To our knowledge, this study is the first to provide information about the occurrence of *E. multilocularis, Toxocara* spp*. and T. gondii* in cat, dog and fox faeces deposited in privately owned kitchen gardens located in a highly endemic area for AE. As an exploratory study, it is based on an opportunistic sampling design leading to uneven sampling regarding villages, sampling periods and sample size per garden. However, despite this and other methodological issues, this study gives an initial insight into the key role kitchen gardens may play in the transmission of foodborne parasites from carnivores to humans.

The use of molecular screening (PCR and qPCR) allowed the identification of the emitter species of faecal samples as well as the detection of the parasite DNA contained in the sample. The proportion of samples usable for molecular analysis (75.2%) was lower than previously described in Knapp et al. [25], where less than 2% of the samples contained PCR inhibitors. However, in Knapp et al. [25], only fox stools were investigated, while in this study we also investigated dog and cat faeces, with cat stools accounting for a very large percentage of the faecal samples with inhibitors. As a high level of calcium is known to alter DNA quality [37], one might assume that cat and dog copro-samples had a higher level of calcium than fox copro-samples due to the high proportion of pet food in these animals’ diet. The correct morphological identification of carnivore stools was high for cat stools (87% of faeces were correctly identified in the field) and fox stools (83% were correctly identified), but was lower for dog stools (63% were correctly identified). These results indicate that identifying copro-material remains difficult, even for specialists, and that confirmation by a second technique is often advisable.

In the majority of previous studies dealing with the detection of foodborne parasites in copro-samples from cat, fox or dog populations, only one faecal sample was collected per individual. In this study, faeces were collected in the field, which means that we could not establish whether multiple samples from the same individual were collected. For this reason, our results have a disadvantage in that they cannot be compared to those from studies on the prevalence of *E. multilocularis* in fox and dog populations (e.g. [12, 52]) or the prevalence of *Toxocara* spp. in fox, dog and cat populations (e.g. [4, 55]). However, they do serve to provide a general view of the contamination risk in kitchen gardens. Indeed, as discussed by Lass et al. [31], the detection of parasite DNA in faeces is evidence of their presence in the environment and indicates a potential risk for humans, even though this alone does not allow the level of risk to be determined – this would require investigating the viability of the infective stage of the parasites and the parasite burden per faeces. In line with Conraths and Deplazes [6], we consider that data from faeces collected in the environment has the advantage of yielding more relevant information for preventing foodborne parasite transmission than investigating prevalence in definitive host populations, since humans run a much higher risk of contamination from contact with the environment than from contact with definitive hosts. Furthermore, such data also have the advantage of allowing hotspots of intensive transmission to be identified, some having already been identified in the transmission of *E. multilocularis* to its intermediate hosts [43] and in the transmission of *Toxocara* spp*.* to humans [35].

Almost one-third of the faeces we collected in kitchen gardens were from fox. This relatively high occurrence of faeces from a wild carnivore in close proximity to humans is not surprising since foxes are known to come close to villages and buildings at night in rural areas [21, 28]. This is likely to explain why *E. multilocularis* DNA was detected close to rural homes in Poland, where foxes approach human settlements in search of food [50]. In our study, 66% of the sampled kitchen gardens were in open access, i.e. easily accessible to canids, and most were near pastures, meadows or forest edges where voles, the main fox prey in the area, are generally abundant [18]. Accessibility and food availability could thus explain the relatively frequent collection of fox faeces in the sampled kitchen gardens. The detection of *E. multilocularis* DNA in almost 35% of the kitchen gardens is thus not surprising since more than 50% of foxes necropsied during the 2001–2005 period were found to carry *E. multilocularis* worms in their intestines [19] and there is no reason to assume that the prevalence of this parasite in the vulpine population was lower during the sampling period of our study. *Echinococcus multilocularis* was also detected in two dog faeces (out of 18) collected in kitchen gardens from two different villages. Given the high reproductive potential of this parasite in fox and dog [22], we can assume that the *E. multilocularis* DNA detected in fox and dog faeces originated from eggs. Nonetheless, the distinction between viable and non-viable eggs would provide valuable information when considering the infection risk. In any case, given the occurrence of fox faeces in kitchen gardens, the occurrence of *E. multilocularis* DNA in these faeces as well as in dog faeces, and the non-homogeneous distribution of faeces testing positive for parasites we observed, the results indicate that fruits and vegetables cultivated in some kitchen gardens could be at risk of exposing consumers to *E. multilocularis*. The detection of *E. multilocularis* DNA in different environmental matrices in a kitchen garden belonging to an AE patient in Poland [30] tends to confirm this hypothesis. Similarly, the contamination of market gardens by feral dog faeces was identified as being probably responsible for numerous cases of human cystic echinococcosis in Jordan [10].

Cat faeces in kitchen gardens were characterised by their abundance, accounting for 58% of the 219 faeces identified from morphological and/or molecular identification. This high occurrence probably resulted both from the relatively high density of the cat population, as compared to the dog and fox populations, and to the selection of kitchen gardens by cats to defecate because they seek to cover their faeces with loose soil. Due to this covering behaviour, the detection of cat faeces in kitchen gardens may have been less exhaustive than the detection of fox and dog faeces. However, the possible underestimation of cat faeces was probably low in the November to February sessions because the soil was generally frozen or wet, deterring cats from burying their faeces. It may have been higher in March and April, when the soil had thawed and some cat faeces may have been deeply buried as gardeners often turn over the soil during this period. Although *E. multilocularis* DNA was detected in almost 7% of the cat faeces collected in kitchen gardens, cats probably play a minor role in the transmission of this parasite since the reproductive potential of *E. multilocularis* is thought to be low in this species [22]. This assumption is supported by the fact that no eggs were found in the 10 out of 321 cat faeces collected in two villages of the study area that tested positive for *E. multilocularis* in a molecular analysis, as well as by the fact that the infected cats originating from these villages carried only immature worms [53]. However, Knapp et al. [27] reported eggs in cat faeces in eastern France, observing entire eggs, with shell and hooks, under the microscope. The role of cat in *E. multilocularis* transmission in terms of the infectivity of eggs warrants further study.

The 33.3% (27.0–48.6) occurrence of *Toxocara* spp. DNA identified by qPCR in cat faeces collected in kitchen gardens is very similar to the 35.7% (31.2–40.1) occurrence of eggs from this parasite detected by the flotation technique on faeces collected in the environment from a stray cat population in Argentina [49]. This similarity may be explained by the fact that in our study, as in that of Sommerfelt et al. [49], only a small percentage of the individuals from the outdoor cat population depositing the faeces collected in kitchen gardens had been dewormed [16]. In contrast, the 11.6% (5.1–21.6) occurrence of *Toxocara* spp. DNA in fox faeces collected in kitchen gardens was lower than the 22.3% (19.9–24.7) occurrence of eggs from this parasite in 1213 fox faeces collected in the field in Belarus and examined with standard flotation techniques [47]. The direct observation of eggs would allow for more reliable assessment of the zoonotic risk related to the detection of *Toxocara* spp. DNA in cat, fox and dog faeces collected in kitchen gardens. Nonetheless, the occurrence of faeces testing positive in our sample suggests that this risk may be significant.

*Toxoplasma gondii* DNA was detected in 1.6% of the cat faeces collected in kitchen gardens; this is in line with the low proportion of individuals found to excrete oocysts in cat populations [11]. *Toxoplasma gondii* was also detected in two dog faeces, which was an unexpected result as canids do not usually excrete *T. gondii* oocysts. *Toxoplasma gondii* DNA detected by qPCR may originate from bradyzoites of infected prey a cat or dog has just ingested, as has been demonstrated experimentally by Poulle et al. [42]. Furthermore, as coprophagy is relatively common in dog [17, 36], the *T. gondii* DNA detected in the two dog faeces may also have resulted from the ingestion of cat faeces with *T. gondii* oocysts*,* as has been suggested to explain the detection of *Toxocara cati* and other atypical parasites in dog faeces [14, 36]. More of a concern could be the detection of *E. multilocularis* and *Toxocara* spp. in dog faeces collected in kitchen gardens; particularly the discovery of two faeces yielding positive qPCR results for these parasites in the same kitchen garden. The fact that the dog that deposited these faeces was confined in this kitchen garden indicates that eggs from this infected individual were shed where fruit and vegetables were grown and could thus present a zoonotic risk for the dog’s owners and any consumers of the garden’s produce.

In conclusion, this study revealed that faecal deposition by cats, foxes and dogs does not appear minor in certain kitchen gardens of northeastern France, nor does the occurrence of foodborne zoonotic parasites in their faeces. In particular, the high occurrence of *E. multilocularis* DNA in fox and dog faeces emphasises the need to prevent access to kitchen gardens by canids to the extent possible in areas where AE is endemic. This could be done through information campaigns to make gardeners aware of the zoonotic threat, by promoting the enclosure of kitchen gardens with fences, by reducing food availability in proximity and by avoiding using kitchen gardens as dog pens. In addition, the deposition of faeces in kitchen gardens by free-roaming cats can lead to a risk of human exposure to *Toxocara* spp. that should not be underestimated [8]. Preventing free-roaming cats from accessing kitchen gardens is difficult as they can easily climb fences, but other recommendations to limit the risk of toxocariasis and toxoplasmosis infections could be promoted, such as washing hands after contact with soil or plants, and cooking or thoroughly washing fruit and vegetables in contact with soil. These recommendations would also be valuable in helping to prevent AE infection.
